# Assessing the power of tag SNPs in the mapping of quantitative trait loci (QTL) with extremal and random samples

**DOI:** 10.1186/1471-2156-6-51

**Published:** 2005-10-19

**Authors:** Kui Zhang, Fengzhu Sun

**Affiliations:** 1 Section on Statistical Genetics, Department of Biostatistics, School of Public Health, University of Alabama at Birmingham, Birmingham, AL 35294, USA; 2 Molecular and Computational Biology Program, Department of Biological Sciences, University of Southern California, Los Angeles, CA 90089, USA

## Abstract

**Background:**

Recent studies have indicated that the human genome could be divided into regions with low haplotype diversity interspersed with regions of high haplotype diversity. In regions of low haplotype diversity, a small fraction of SNPs (tag SNPs) are sufficient to account for most of the haplotype diversity of the human genome. These tag SNPs can be extremely useful for testing the association of a marker locus with a qualitative or quantitative trait locus in that it may not be necessary to genotype all the SNPs. When tag SNPs are used to reduce the genotyping effort in association studies, it is important to know how much power is lost. It is also important to know how much power is gained when tag SNPs instead of the same number of randomly chosen SNPs are used.

**Results:**

We design a simulation study to tackle these problems for a variety of quantitative association tests using either case-parent samples or unrelated population samples. First, the samples are generated based on the quantitative trait model with the assumption of either an extremal sampling scheme or a random sampling scheme. Second, a small number of samples are selected to determine the haplotype blocks and the tag SNPs. Third, the statistical power of the tests is evaluated using four kinds of data: (1) all the SNPs and the corresponding haplotypes, (2) the tag SNPs and the corresponding haplotypes, (3) the same number of evenly spaced SNPs with minor allele frequency greater than a threshold and the corresponding haplotypes, (4) the same number of randomly chosen SNPs and their corresponding haplotypes.

**Conclusion:**

Our results suggest that in most situations genotyping efforts can be significantly reduced by using tag SNPs for mapping the QTL in association studies without much loss of power, which is consistent with previous studies on association mapping of qualitative traits. For all situations considered, two-locus haplotype analysis using tag SNPs are more powerful than those using the same number of randomly selected SNPs, but the degree of such power differences depends upon the sampling scheme and the population history.

## Background

Single-nucleotide polymorphism (SNP) markers are preferred over microsatellite markers in association studies because of their high abundance, low mutation rate, and suitability for high-throughput genotyping. The genome-wide association studies on dissection of human complex traits need to screen a large number of SNPs. However, it is prohibitively expensive to genotype all SNPs in an association study with the throughput of current technologies. Judicial selection of SNPs for association studies is therefore of paramount importance. The observation that the human genome can be divided into regions of high linkage disequilibrium (LD) with limited haplotype diversity interspersed with regions of low LD suggests one way of doing this. The regions with high LD are referred to as blocks in the literature. One of the objectives in the Human HapMap project is to describe the set of haplotype blocks and the SNPs that tag them.

Many methods have recently been developed for haplotype block partitioning and tag SNP selection. Available methods can be classified into two groups – block-dependent methods and block-free methods – although all of them are based on LD patterns of the human genome. The first group of methods relies on haplotype diversity or pair-wise LD measures such as D' to first partition the haplotypes into blocks and then select tag SNPs in each resulting block (e.g., [1-5]). The other methods select tag SNPs directly in accordance with LD patterns (e.g., [6-8]) or through comprehensive power computations (e.g., [9-11]) across the human genome. However, it is still not clear which method should be used in tag SNP identification. Here, we concentrate on two different methods. One is a variant of the first group of methods, which involves partitioning the haplotypes into blocks to minimize the total number of tag SNPs over a region of interest or the whole genome [4,5,12,13]. With this method, we expect to reduce the genotyping effort as much as possible. The other method selects tag SNPs based on pair-wise LD measure *r*^2 ^[6], where for each SNP the maximum *r*^2 ^between this SNP and tag SNPs must be greater than a pre-specified threshold.

The general procedure for using tag SNPs in association studies can be described as follows. First, a small number of samples (e.g., 40~50 individuals) are genotyped using a very dense SNP map. Second, a method or an algorithm is applied to obtain the set of tag SNPs. Third, a large number of samples is genotyped at only the tag SNP marker loci. Fourth, association tests of the SNPs with a qualitative or quantitative trait of interest are conducted using all the genotyped samples at tag SNP marker loci. The above approach can significantly reduce the genotyping effort [14], but it also causes loss in statistical power for association studies. There are two key questions. First, how much power will be lost when tag SNPs instead of all the SNPs are used. Second, how much power will be gained when tag SNPs instead of the same number of randomly chosen SNPs are used if only a given number of SNPs can be genotyped due to limited resources.

Several studies have examined these problems for association mapping of qualitative traits. Zhang et al. [12,13] studied this problem for qualitative traits with simulations and found that the loss of power was moderate – certainly much smaller than if an equivalent number of markers had been chosen randomly. Thompson et al. [15] used similar simulation procedures and observed similar results. Zhai et al. [16] did a similar simulation study but chose a set of evenly spaced SNPs with minor allele frequency greater than a threshold. They found that the power to detect association with evenly distributed SNPs can be almost the same as the power to detect association with tag SNPs, and sometimes even better. However, this study has several limitations. Zhai et al. [16] did not use haplotype based methods, which can be more powerful than methods based on individual marker when the disease susceptibility SNP is not included in the set of SNPs used for mapping. The number of tag SNPs is usually much smaller than the number of all SNPs, and the tag SNPs have a much sparse map, which can be favorable for haplotype analysis. This was clearly shown in the power comparison of Zhang et al. [12,13]. Also, the highest power occurred when the markers and the disease gene had similar allele frequencies [17,18]. However, it is very difficult to know the disease allele frequency in advance. Thus, the threshold that should be used to select SNP markers is problematic. Zhang et al. [18] studied power using tag SNPs identified by haplotype diversity and found that tag SNPs may not be efficient when the allele frequency at the marker locus is much different from the allele frequency at the disease locus. Other studies have investigated the power issue theoretically, which may not account for the complexities and heterogeneities in LD mapping of disease genes, and thus their conclusions cannot be readily extended to comprehensive haplotype-based methods [6,10,19]. Furthermore, most studies involving assessment of such power loss with tag SNPs have focused on qualitative traits using either simulations or real data sets [6,12,13,15,16]. The exception is Zhang et al. [18], who studied quantitative traits. However, they did not consider the analysis based on haplotypes. In this paper, we assess the loss of power to detect quantitative trait loci using tag SNPs through extensive simulations. One major difference between this study and previous studies is the use of haplotypes to study quantitative traits.

## Methods

### The coalescent with recombination

To carry out our study, we first simulated a large number of haplotypes consisting of a large number of consecutive SNPs across a genomic region. The simulation procedure and corresponding parameters are similar to simulations conducted in our previous studies [12,13]. Specifically, we used the coalescent process with recombination [20-22] to construct haplotype populations. The genealogies of haplotypes were generated with a population recombination rate *r *over the region of interest, and they are denoted by [0, 1] for easy presentation. Once the ancestral relationship between haplotypes has been simulated, mutations are added onto the genealogies to generate SNPs with a population mutation rate *θ *according to the infinite-many-site mutation model. The infinite-many-site mutation model assumes that mutations occur uniformly in [0, 1] and that a new mutation creates a new SNP that does not already exist in the population – i.e., recurrent mutations are not allowed.

It is well known that recombination hot and cold spots can give rise to discrete haplotype block-like structure [23,24]. Studies from both empirical data and simulations also suggested that haplotype blocks may be created due to genetic drift [25,26]. To accommodate such features of human evolution in our simulations, we employed four different population models to generate haplotypes. For the first population model, we assumed a constant population size with a uniform recombination rate. We set both *r *and *θ *to 200, which correspond to simulating a genomic region of about 200 kb [27]. We simulated the second haplotype population with recent population expansion but with the assumption of a uniformly distributed recombination rate. Again, both the recombination rate and the mutation rate were set at 200. We assumed that the population was constant at size 10,000 for a very long time and that it began growing exponentially until it reached the present population size of 10^7 ^from 1,500 generations ago. We also generated two haplotype populations with varied schemes of recombination hot spots. Both the mutation rate and the background recombination rate were set at 200 in this situation. For the third population model, two regions [0.20, 0.30] and [0.70, 0.80] were selected, with recombination rates 15 times higher than the background recombination rate. For the fourth population, one region [0.40, 0.60] was selected, with a recombination rate 15 times higher than the background recombination rate. The first two population models have been used in previous studies [12,13,16]. As we will describe below, the simulated disease susceptibility loci were positioned within the region [0.40, 0.60]. The two additional populations allow us to thoroughly assess the effect of recombination hot spots on the power loss because the disease susceptibility gene is not in recombination hot spot regions for the third population, while it is in a recombination hot spot region for the fourth population. For simplicity, we refer to these population models as P1, P2, P3, and P4, respectively, in the rest of this paper.

We simulated a quantitative trait locus with the frequency of the allele corresponding to the high trait value in a designated range. Here, we considered three different scenarios corresponding to rare, moderate, and common alleles for the high trait values, respectively. For each set of haplotypes, we chose a marker locus as the candidate trait locus if it satisfied two conditions: (1) the frequency of the minor allele is in a designated range, and (2) the position of the trait locus is between 0.40 and 0.60. The first condition restricts the variant allele frequency, and the second condition ensures that the candidate trait locus is approximately in the middle of the region of interest. If no such marker loci exist, this data set was discarded. If several marker loci satisfy these conditions in a data set, the marker locus closest to 0.50 was chosen as the quantitative trait locus. Once the candidate trait locus has been determined, the marker loci were selected sequentially from the left to the right along the chromosome based on the following conditions. (1) The frequency of the minor allele is at least 5%. (2) The distance between any two adjacent marker loci, including the candidate trait locus, is not less than a threshold. In this study, we set the threshold at 0.005. Because the length of the simulated genomic region is about 200 kb, the distance between two adjacent markers is at least 200*0.005 = 1 kb, resulting in at most 200 markers. In addition, the trait locus is not one of the marker loci used in mapping and is away at least 0.005 from the closest marker locus. The haplotypes at these marker loci and the trait locus were retained for further analysis.

### The quantitative trait models

Based on the set of haplotypes generated above and a given quantitative trait model, we generated parents-offspring samples or population samples using either random sampling or extremal sampling. We considered the following widely used quantitative trait model at the candidate trait locus:

*Q*_*i *_= *μ *+ *aA*_*i *_+ *dD*_*i *_+ *ε*_*i*_,     (1)

where *Q*_*i *_is the trait value; *A*_*i *_and *D*_*i *_are the additive and dominant genotypic scores, respectively; and *ε*_*i *_is a normal random variable with mean 0 and variance 1 and is independent of the genotype.*A*_*i *_takes the values 1, 0, and -1, and *D*_*i *_takes the values 0, 1, and 0 for genotypes *MM*, *Mm *and *mm*, respectively, in which *M *is the allele corresponding to the high trait value. The additive genetic variance attributable to the locus is , the dominant genetic variance is , and the total genetic variance is , where *p *is the frequency of the allele corresponding to the high trait value at the trait locus and *q *= 1 - *p*. The broad-sense heritability attributable to the locus is computed by [28,29].

Here, we only considered the additive model (*d *= 0). For a given frequency of the allele corresponding to high trait value *p*, and the broad-sense heritability *H*^2^, we calculated the value of *a*. In this paper, we let *μ *= 0 and *H*^2 ^= 20%. For random sampling, we generated 600 family samples consisting of unrelated individuals with their parents and 300 population samples of unrelated individuals based on the given quantitative trait model. For the family samples in extremal sampling, we chose 125 individuals with trait values in the top 20% of the population distribution of the trait values together with their parents and 125 individuals with trait values in the lower 20% of the population distribution of the trait values together with their parents. For the population samples in the extremal sampling, we selected 75 individuals with trait values in the upper 20% as cases and 75 individuals with trait values in the lower 20% as controls. We found that the power using these sample sizes for most methods is in an appropriate range under all situations considered, and thus meaningful comparisons can be made.

### Algorithms for tag SNP selection and random SNP selection

Many methods have recently been developed for haplotype block partitioning and tag SNP selection. Here, we mainly focus on two methods. The first method is block-dependent, in which a dynamic programming algorithm is used to find the optimal block partition to minimize the total number of tag SNPs [5,12]. We followed commonly used definitions of blocks and tag SNPs [4,5] in our simulation. We defined blocks as in Patil et al. [4], where at least *α *percentage of observed or inferred haplotypes must be common haplotypes. Common haplotypes are those with frequencies greater than a threshold *β*. We defined tag SNPs within a block as the minimal set of SNPs that can distinguish *α *percentage of all observed or inferred haplotypes. We fixed *α *and *β *as 0.80 and 0.05, respectively. The second method is a block-free method based on LD measure *r*^2 ^[6]. Because the statistical power of association studies is proportional to the value of *r*^2 ^[30], this method has become popular in recent studies. Here, for any given subset of SNPs, all pair-wise *r*^2 ^values between the SNPs in this subset and the SNPs not in this subset were calculated. For a given SNP not in the subset, we took the maximum value of *r*^2 ^as its individual prediction power. The minimum value over all of the SNPs was taken as the overall prediction power. The minimum set of SNPs with prediction power exceeding a pre-specified threshold, *γ*, was considered as a set of tag SNPs. We adapted the greedy algorithm developed by Carlson et al. [6] to select the set of tag SNPs. For comparison, we choose an appropriate *γ *that enables the same number of tag SNPs for two different algorithms.

For power comparison, Zhang et al. [12,13] used a set of SNPs chosen uniformly at random among all SNPs. Zhai et al. [16] argued that researchers would prefer a set of evenly spaced SNPs with minor allele frequencies greater than a threshold if no prior knowledge of these SNPs was available in real studies, and they conducted the power comparison of association studies between them and tag SNPs. Here, we chose the same number of SNPs using both methods. The threshold for the minor allele frequency, *t*, was set to 0.05, 0.10, 0.15, and 0.20, respectively.

### Tests of association of quantitative trait locus (QTL) by linkage disequilibrium

Linkage disequilibrium mapping studies for QTL typically use either family samples or unrelated individuals. When family data are used, the transmission/disequilibrium test (TDT) for quantitative traits can be used to test for linkage or association [32-35]. In this study, we assumed that we had *n *families with two parents and one offspring in each family, and we used the statistic TDTQ [33,34]. Many methods have been developed for mapping quantitative trait loci using unrelated population individuals [28,29,36]. Here, we employed the regression method to test whether there is any association between a marker locus and a QTL. Suppose we have the genotypes and the trait value of *n *individuals. The test of association can be implemented based on the standard linear regression model, as in equation (1). The null hypothesis is *a *= *d *= *0*.

The QTL mapping using haplotype data is of great interest. Thus, we also implemented the haplotype-based method developed by Dudbridge [37] and Zaykin et al. [38]. The first method is an extension of classical TDT, and the second is based on regression analysis. Both methods estimate haplotypes and their frequencies using the EM algorithm and can account for the uncertainty of haplotype frequencies.

## Results

We generated 20 sets of 2,000 haplotypes using the coalescent program with population models from P1 to P4, respectively. The QTL and the marker loci used for mapping were determined by the approach described in the Methods section. In summary, the number of markers used for mapping varies from 131 to 156. For the rare QTL, the frequency of the allele corresponding to high trait values varies from 0.040 to 0.060. For the moderate QTL, the frequency of the allele corresponding to high trait values varies from 0.125 to 0.175. For the common QTL, The frequency of the allele corresponding to high trait values varies from 0.270 to 0.329. The position of the QTL varies from 0.443 to 0.583.

These sets of haplotypes were then used to construct samples with quantitative traits. For each set of haplotypes, we generated 50 replicates of parent-offspring samples or population samples using either the random sampling scheme or the extremal sampling scheme described in the Methods section. We thus had a total of 1,000 replicates for each sampling scheme and each population. For each set of family samples, 20 pairs of parents (i.e., 80 haplotypes) were randomly selected to obtain the haplotype block partitions and tag SNPs. For each set of population samples, we chose 40 individuals (i.e., 80 haplotypes) as tagged samples. Several studies have shown that such a number of individuals can give similar block partitions and tag SNPs as a larger number of samples [12,15,26]. We calculated the test statistics based on individual SNPs and two-locus haplotypes, and we adjusted the *p*-values over all the markers using the Bonferroni correction. Table 1 summarizes the test methods compared in this study. The power of each test was conducted using several different kinds of data with type I error of 0.05: (1) all the SNPs, (2) the tag SNPs, (3) the same number of evenly spaced SNPs with minor allele frequency greater than a threshold, and (4) the same number of randomly chosen SNPs as the number of tag SNPs.

**Table 1:** Summary of methods used for power comparison.

**Test Methods**	**Description**
**TDT-R-SNP**	Random sampling, marker-by-marker analysis for family samples
**TDT-R-HAP**	Random sampling, two-locus haplotype analysis for family samples
**POP-R-SNP**	Random sampling, marker-by-marker analysis for population samples
**POP-R-HAP**	Random sampling, two-locus haplotype analysis for population samples
**TDT-E-SNP**	Extremal sampling, marker-by-marker analysis for family samples
**TDT-E-HAP**	Extremal sampling, two-locus haplotype analysis for family samples
**POP-E-SNP**	Extremal sampling, marker-by-marker analysis for population samples
**POP-E-HAP**	Extremal sampling, two-locus haplotype analysis for population samples

### Power comparisons

**Figure 1:**
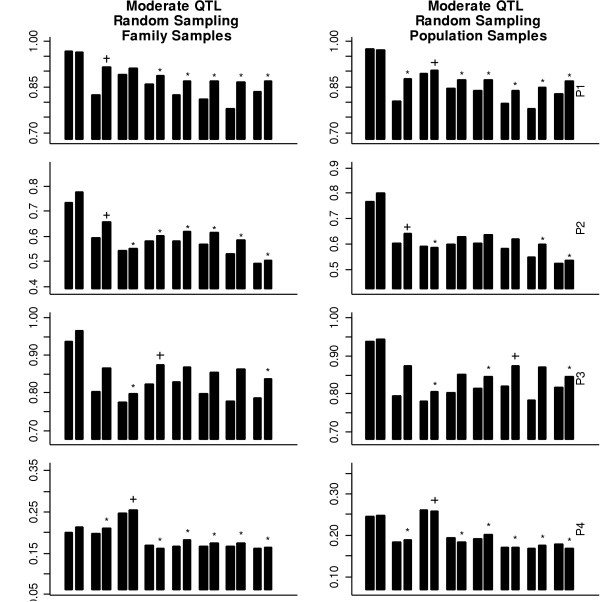
The power using SNPs with a random sampling scheme for different population models. The power is obtained using single-SNP and two-locus haplotype data based on 1,000 simulations with a moderate QTL. In each bin, the figure shows the power based on marker-by-marker analysis and two-locus haplotype analysis (from left to right). Between bins, it shows the power using (from *left *to *right*): (1) all SNPs; (2) the tag SNPs identified using the haplotype diversity [4]; (3) the tag SNPs identified using *r*^2 ^[6]; (4) the evenly spaced SNPs with minor allele frequencies greater than 0.05; (5) the evenly spaced SNPs with minor allele frequencies greater than 0.10; (6) the evenly spaced SNPs with minor allele frequencies greater than 0.15; (7) the evenly spaced SNPs with minor allele frequencies greater than 0.05; and (8) the randomly selected SNPs. In each graph, the method having the highest power based on two-locus haplotype analysis is indicated with the "+" sign. The methods having power significantly lower than the highest one (one-sided chi-square test with 0.05 type I error rate) are indicated with the "*" sign.

**Figure 2:**
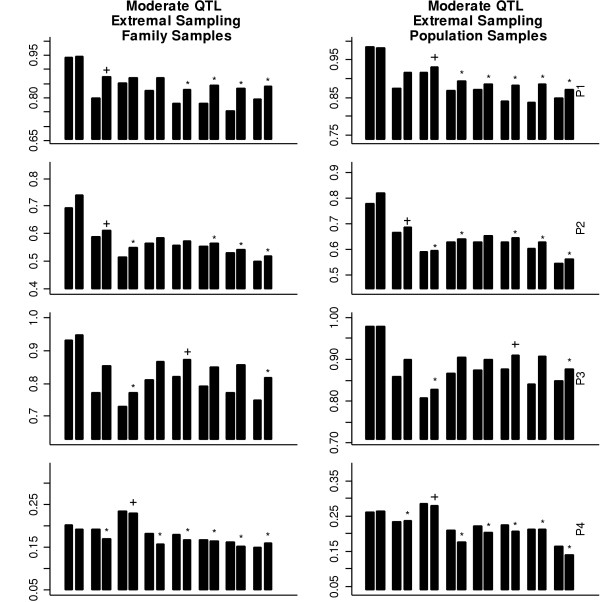
The power using SNPs with an extremal sampling scheme for different population models. The power is obtained using single-SNP and two-locus haplotype data based on 1,000 simulations with a moderate QTL. The bars in each bin and the symbols ("+" and "*") have the same meaning with those in Figure 1.

Here, we describe the results from our power study using the above methods with a significance level of 0.05. On average, 36, 44, 43, and 42 tag SNPs were selected for population models from P1 to P4, respectively. As expected, more tag SNPs were needed with the inclusion of the recombination hot spots and the population expansion. For the moderate QTL, the power results for the different testing methods and populations with the random sampling scheme and the extremal sampling scheme are shown in Figure 1 and Figure 2, respectively. Several general conclusions emerge from these figures. First, except for the degree of the power differences among the tests, Figure 1 and Figure 2 show very similar patterns, indicating that the conclusions drawn based on the random sampling scheme can be generally applicable to the situation involving the extremal sampling scheme. Second, the tests based on family samples and population samples also have similar power patterns. Third, although the power to detect the QTL based on two-locus haplotypes is generally higher than the power based on marker-by-marker analysis because of the exclusion of the QTL in the analysis. Such a gain in power depends not only upon population models but also on the methods for tag SNPs selection. Fourth, population models used in the simulation substantially affect the power patterns using the tag SNPs, the evenly spaced SNPs, and the randomly selected SNPs. Population models also affect the performance of tag SNPs. Because the power for detecting association is generally very low in population P4, we will only compare our results in the first three populations (P1, P2, and P3).

For individual marker analysis, there are no clear patterns to indicate which approach for tag SNP selection performs the best. In population P1, the tag SNPs identified using *r*^2 ^perform the best. In population P2, the tag SNPs identified using haplotype diversity outperform the tag SNPs identified using other methods. In population P3, the maximum power among the evenly spaced SNPs is the highest. These differences can be quite significant. The findings in this study are consistent with previous studies [16,18].

We emphasize that the idea behind haplotype tagging is to account for most haplotype diversity using the smallest number of tag SNPs and then to do haplotype-based analysis, not individual marker analysis. Indeed, haplotype-based analysis performs similarly in high-LD regions and outperforms in low-LD regions compared with individual marker analysis. Note that the QTL is excluded from our analysis. If the QTL is one of the marker loci analyzed, individual marker analysis can be more powerful than haplotype analysis. We envision that the chance of having the QTL in the marker set is low, and thus we suggest using haplotype analysis in real studies. Next, we concentrate on haplotype analysis. In population P1, with high LD, the performances of the three methods are similar. In population P2, with medium LD, the tag SNPs identified based on haplotype diversity perform significantly better than those selected using the other two approaches. In population P3, with regions of low LD and regions of high LD, the tag SNPs identified based on haplotype diversity perform similarly to the evenly spaced SNPs and perform significantly better than the tag SNPs selected based on *r*^2^. In all but one case, the tag SNPs identified based on haplotype diversity perform the best or are not significantly different from the best-performing ones.

**Figure 3:**
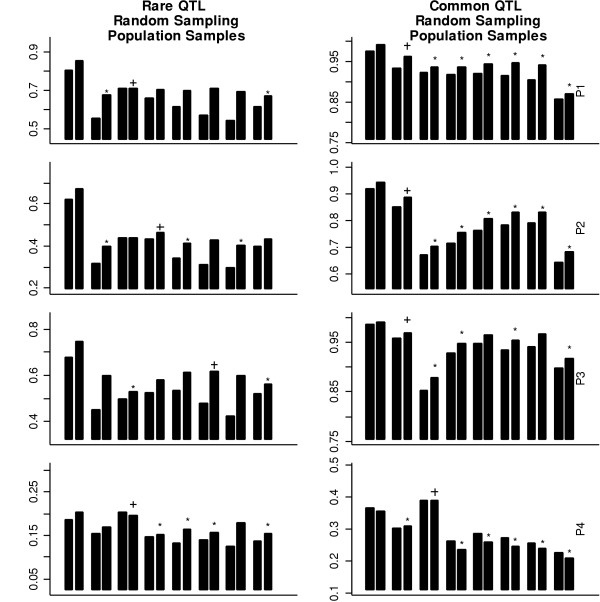
The power using SNPs with random sampling of population samples for different population models. The power is obtained using single-SNP and two-locus haplotype data based on 1,000 simulations with a rare and a common QTL, respectively. The bars in each bin and the symbols ("+" and "*") have the same meaning with those in Figure 1.

For rare and common QTLs, the power patterns for random or extremal sampling, population or family sampling are also similar. Therefore, we only present the results based on random sampling of population samples in Figure 3. For rare QTLs and individual marker analysis, the tag SNPs identified based on haplotype diversity perform poorly and have smaller power even than the randomly selected SNPs in populations P1, P2, and P3. This is expected because the method based on haplotype diversity tends to select more common SNPs, while the rare SNPs have more power to detect the QTL at this situation. The tag SNPs selected based on *r*^2 ^have the highest power in populations P1 and P2 and have power comparable with the evenly spaced SNPs in population P3. The evenly spaced SNPs, with minor allele frequencies close to the frequency of the high-risk allele at QTLs, have the highest power in population P3 and have power comparable with the tag SNPs selected based on *r*^2 ^in populations P1 and P2. However, the evenly spaced SNPs with minor allele frequencies greater than 0.15 generally perform poorly and have the least power in all populations.

For common QTLs and individual marker analysis, the performances of the three methods are similar in population P1. The tag SNPs identified based on haplotype diversity performs significantly better than the other two approaches in population P2. In population P3, the tag SNPs identified based on haplotype diversity perform similarly to the evenly spaced SNPs and significantly better than the tag SNPs selected based on *r*^2^.

For rare QTLs and two-locus haplotype analysis, the tag SNPs identified based on haplotype diversity are significantly less powerful than the tag SNPs selected based on *r*^2 ^or the evenly spaced SNPs in populations P1 and P2 (a one-sided chi-square test with a 0.05 type I error rate) but have power comparable to the evenly spaced SNPs in population P3. The tag SNPs selected based on *r*^2 ^perform similarly to the evenly spaced SNPs in populations P1 and P2 but are significantly less powerful than the other two methods in population P3. For common QTLs and two-locus haplotype analysis, the power of tag SNPs identified based on haplotype diversity is the highest and is significantly greater than the power of the tag SNPs identified based on *r*^2 ^and the evenly spaced SNPs in populations P1, P2, and P3. Again, the tag SNPs identified based on *r*^2 ^have less power than the evenly spaced SNPs in populations P1, P2, and P3.

These power patterns can arise for several possible reasons. First, SNPs close to the QTL generally have more power. Therefore, one method will be more powerful than the other methods if it can choose more SNPs around the QTL than the other method methods. As an example, the QTL was positioned far away from two designated recombination hot spot regions for population model P3. The methods based on the haplotype diversity [4] and *r*^2 ^[6] select more SNPs in two recombination regions but fewer SNPs around the QTL than the number of evenly spaced SNPs, resulting in less power to detect the QTL. In contrast, more tag SNPs were selected around the QTL for population model P4, and in this situation the power to detect the QTL using the tag SNPs is higher than the power when the same number of evenly spaced SNPs is used. Second, it has been suggested that both the allele frequencies of marker loci and the QTL affect the power in association studies. The power generally achieves its maximum when the minor allele frequency of SNPs is close to the frequency of the disease allele for individual marker analysis [17]. This is very clear when we compare the power of individual marker analysis based on rare QTLs and common QTLs. Here, we recorded the minor allele frequency in each of 1,000 replicates.

**Figure 4:**
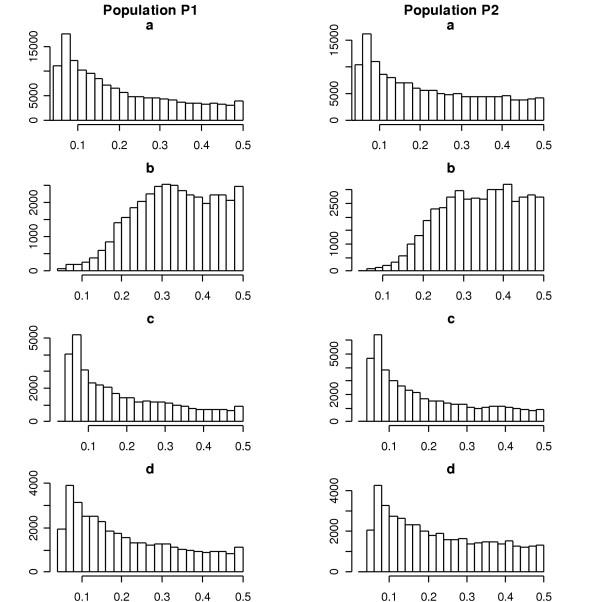
The histogram of the minor allele frequencies for those selected SNPs for population models P1 and P2 with random sampling of family samples and moderate QTLs. In each column, (a) represents all SNPs (b) the tag SNPs identified using the haplotype diversity [4], (c) the tag SNPs identified using *r*^2 ^[6], and (d) the evenly spaced SNPs with minor allele frequencies greater than 0.05, respectively.

Figure 4 shows the histogram of the minor allele frequencies for those selected SNPs for population models P1 and P2 with random sampling of family samples and moderate QTLs. It can be seen that the distributions of minor allele frequencies for the tag SNPs identified by *r*^2 ^and the evenly-spaced SNPs with minor allele frequencies greater than 0.05 are similar in Figure 4, and that they have a shape similar to the distribution of minor allele frequencies for all of the SNPs. On the other hand, the distribution of minor allele frequencies for the tag SNPs identified using haplotype diversity is different from others. This tag SNP selection method tends to choose more common SNPs in order to characterize the common haplotypes using as few tag SNPs as possible within each block.

## Discussion

Genome-wide association studies based on linkage disequilibrium patterns play a central role in localizing genetic variation responsible for common human diseases and traits. Recently, several studies have revealed a block-like structure across the human genome. Understanding this block-like structure is essential for the current effort. It is important to develop methods for locating the haplotype block structure and the corresponding tag SNPs as well as to understand the usefulness and limitations of tag SNPs in association studies for QTL mapping. In this paper, we used Monte Carlo simulations to assess the power loss when the tag SNPs instead of all of the SNPs are used to detect the QTL in association studies. We also compared the power using tag SNPs and the power using the same number of evenly-spaced SNPs and randomly chosen SNPs. This is one of few studies to assess the power using tag SNPs to detect the QTL. Our results confirmed some conclusions from previous studies with qualitative traits and produced some novel findings. We showed that there are no clear winners for the three tag SNP selection methods studied in this paper based on individual marker analysis. For two-locus haplotype analysis and QTLs with moderate to high minor allele frequency, the tag SNPs identified based on haplotype diversity are comparable to the best approach in almost all of the situations examined. However, the power of the tag SNPs selected based on *r*^2 ^can be significantly lower than the power of the best methods. On the other hand, the evenly spaced SNPs perform quite well in most situations if we know the allele frequency of the QTL, but they can express relatively low power when the allele of the QTL is incorrectly specified. For QTLs with low minor allele frequency, the power using tag SNPs identified based on haplotype diversity can be much lower than the power using the other two approaches.

Several possible improvements in future studies can be carried out using more sophisticated simulation strategies. In this paper, we used the coalescent process to simulate the haplotypes because the coalescent theory captures the essentials of the population genetic data. It also allows us to explore the effects of some key factors, such as the mutation rate, the recombination rate, and the population expansion. In addition to the populations simulated with constant population size and uniformly distributed mutation and recombination rates, we also generated haplotypes with the inclusion of recombination hot spots and rapid population expansion. Our results show that the population history has a substantial effect on the usage of tag SNPs in association studies. However, simulations based on the coalescent model may fail to capture some features of human evolution as found in real data sets. Therefore, simulations based on real data sets would be desirable. In this paper, we considered QTLs with low, moderate, and high minor allele frequencies and it was assumed that they are positioned at the center of region of interest. However, this information generally remains unknown in real studies. A possible approach would be to consider each SNP as a potential QTL, then compare the average power for all the SNPs or the average power based on different ranges of minor allele frequencies. In this paper, the samples used for tag SNP selection are a subset of the samples used to detect QTL. This strategy is different from the HapMap project, in which tag SNPs are identified by a set of samples and then can be used in virtually any studies based on the same population. However, it is far from obvious that tag SNPs chosen in this way will be the best ones for mapping genes in another sample, and there is no assessment of such consequences. Given that the Human HapMap project is nearly complete and that many researchers have attempted to use tag SNPs for their disease-mapping studies, it is clearly important to develop sophisticated simulation plans to evaluate the effectiveness of such a design.

In this paper, we presented our simulation results based on relatively high broad-sense heritability (*H*^2 ^= 20%). It maybe is too high for many QTLs. We also simulated QTLs with broad-sense heritability *H*^2 ^= 5%. In order that the power is relatively high (about 0.70–0.80 using all the SNPs) for meaningful comparisons, a larger sample size is required. Detailed results are provided as supporting materials (Supplemental Figure 1 and 2 [see additional file 1]). The power patterns are similar to those presented in Figure 1 to 4. In our simulations, we used the simple QTL model (a single main QTL with the additive effect) and the simple statistical methods for detecting associations (e.g., TDT and the regression analysis). Nonetheless, our simulations are still valid for many QTLs as long as each of them has the detectable marginal effect. New simulation designs are needed to investigate the effectiveness of tag SNPs in detecting those QTLs with only interactions but no marginal effects. In this study, we also constrained our simulations on a homologous population. The presence of sub-population structures in studied samples can greatly complicate the analysis, not only because it can result false association but also the blocks and tag SNPs depend on the specific populations [39,40]. A possible way around this problem is to first use unrelated SNPs to divide a general population into several homogeneous populations [41], and then obtain the haplotype block partitions and the tag SNPs and conduct the association analysis for each population.

In this study, we concentrated on two tag SNP selection methods. They can represent two distinct groups of methods. The first method is block-dependent and is based on haplotype diversity [4]. The second method is block-free and is solely based on pair-wise LD measure *r*^2 ^[6]. Our results have important implications for association studies in that we found that these two methods perform differently for different population models. There are also many other methods for tag SNP selection, but only a few of them have been evaluated in previous studies. In addition, many factors, including population structure and history [39,40], marker allele frequency [12,42], marker density [12,40,42], and number of samples [13,15,26], can affect the selection of tag SNPs and their performances in association studies. It still remains unclear which method should be used in tag SNP selection for association studies, but we believe no method will perform best under all situations. Thus, it is important to determine which method is better under certain conditions in future studies.

Finally, we would like to emphasize that any method for tag SNP selection must be combined with existing biological knowledge. For example, if two adjacent SNPs are in complete LD with similar minor allele frequencies, the methods based on pair-wise LD may only choose one of them as a tag SNP. If both of them have been suggested by the previous biological knowledge to be important, there is no reason both of them should not be included in the set of tag SNPs. The best way may be to combine several methods to come up with a "consensus set" of tag SNPs with biologically important SNPs.

## Conclusion

In this paper, we studied the power of tag SNPs to detect the QTL using extensive Monte Carlo simulations. Our study confirmed some conclusions from previous studies with qualitative traits and produced some novel findings, which have important implications in designing optimal association studies using tag SNPs. First, the use of tag SNPs can significantly reduces the genotyping effort without much loss of power in most situations. Second, two-locus haplotype analysis using tag SNPs are more powerful than those using the same number of randomly selected SNPs. Third, among several methods for tag SNP selection compared in this paper, there is no single method that outperforms the others in all situations. Fourth, the population structure and history and the allele frequency at the disease locus have substantial effects on the usage of tag SNPs in association studies. The effect of other factors, such as marker allele frequency and marker density, on the power of tag SNP selected by many other methods still remains unclear and needs further investigation.

## Authors' contributions

Kui Zhang participated in the design of the simulation studies, conducted the simulations, performed the statistical analysis, interoperated the results, and drafted the paper. Fengzhu Sun participated in the design of the simulation studies, interpreted the results, and helped to draft the paper. All authors read and approved the final manuscript.

## Supplementary Material

Additional file 1 this Microsoft Word file contains two supplemental figures (supplemental figure 1 and 2) and their legends. These figures present the power results based on the heritability of 5%. Click here for file
