# Supplementary material for: Assessing the power of tag SNPs in the mapping of quantitative trait loci (QTL) with extremal and random samples
Source: BMC Genet. 2005 Oct 19;6:51. doi: 10.1186/1471-2156-6-51 (PMC1274312; doi:10.1186/1471-2156-6-51)
Supplement: Additional file 1 — this Microsoft Word file contains two supplemental figures (supplemental figure 1 and 2) and their legends. These figures present the power results based on the heritability of 5%. [file 1471-2156-6-51-S1.doc]

**Supplemental Figure 1.**

**Supplemental Figure 2.**

Legends of Supplemental Figure 1 and 2.

The power using SNPs with extremal sampling of family or population samples for different population models and different frequencies of the high risk allele with heritability$H^{2}=5\%$. The power is obtained using individual-SNP and two-locus haplotype data based on 1,000 simulations for a moderate (Figure 1), a rare (Figure 2), and a common QTL (Figure 2), respectively.

In each bin, the figure shows the power based on individual-SNP analysis and two-locus haplotype analysis (from left to right). Between bins, it shows the power using (from *left* to *right*): (1) all SNPs; (2) the tag SNPs identified using the haplotype diversity [4]; (3) the tag SNPs identified using $r^{2}$ [6]; (4) the evenly spaced SNPs with minor allele frequencies greater than 0.05; (5) the evenly spaced SNPs with minor allele frequencies greater than 0.10; (6) the evenly spaced SNPs with minor allele frequencies greater than 0.15; (7) the evenly spaced SNPs with minor allele frequencies greater than 0.05; and (8) the randomly selected SNPs.

In each graph, the method having the highest power based on two-locus haplotype analysis is indicated with the “+” sign. The methods having power significantly lower than the highest one (one-sided chi-square test with 0.05 type I error rate) are indicated with the “*” sign.

In this study low heritability $H^{2}=5\%$ was used in the simulation. Large sample sizes were used to ensure that the power for all methods is in an appropriate range to be used in comparison. Specifically, for rare QTLs, a total of 1,000 trios and 600 individuals were used in simulations. For moderate to common QTLs, a total of 1,000 trios and 600 individuals were used in simulations with the population models P2 and P4, while a total of 500 trios and 300 individuals were used in simulations with the population models P1 and P3.

From these two figures, we can find that the patterns of power are very similar with those in Figure 2 and 3 in the main text, although the absolute values of the power vary in different figures.
